# *“What Do You Need?”* Formative Research to Develop a Comprehensive Maternal Needs Assessment Tool for Infant and Young Child Nourishment and Care in the United States

**DOI:** 10.3390/nu17243825

**Published:** 2025-12-06

**Authors:** Mercy Eloho Sosanya, Laura Birgit Mueller, Caleb Martin, Jennifer L. Temple

**Affiliations:** Department of Exercise and Nutrition Sciences, School of Public Health and Health Professions, University at Buffalo, Buffalo, NY 14214, USA

**Keywords:** Maternal Needs Assessment, infant and young child feeding (IYCF), breastfeeding, child development, maternal mental health, psychosocial wellbeing, institutional barriers, work–family balance, digital health information, mixed methods

## Abstract

**Background/Objectives:** Despite substantial healthcare spending, U.S. mothers encounter fragmented support systems for infant feeding, care, and maternal mental health. While existing needs assessment instruments target parents of ill or preterm infants, no validated tool captures the full range of informational, psychosocial, and structural needs among mothers of healthy, full-term infants. This formative mixed-methods study sought to identify and prioritize maternal needs across multiple socioecological levels to guide the development of a comprehensive Maternal Needs Assessment Tool (MNAT). **Methods:** Guided by the socioecological model, six virtual focus groups were conducted with U.S. mothers of healthy infants < 2 years (analytic sample = 28). Thematic analysis in ATLAS.ti (Version 25) identified key needs, which informed the creation of a 10-domain Maternal Needs Assessment Ranking Questionnaire (MNARQ). Participants (*n* = 22) rated each domain’s importance on a five-point scale; weighted mean ranks were calculated in SPSS (Version 30). **Results:** Seven overarching themes across ten domains emerged: infant and young child feeding and care, maternal psychosocial wellbeing, parenting knowledge and skills, interpersonal and community support, institutional assistance, and work-policy environments. The highest-ranked domains of need were complementary feeding, child development, care and health, social norms, networking and support, maternal mental health, and breastfeeding guidance. Mothers described pervasive informational confusion, inadequate professional and peer support, and institutional barriers such as limited postpartum follow-up, inflexible daycare policies, and WIC hurdles in formula substitution for infants with allergies. **Conclusions:** Maternal needs encompass intersecting personal, social, and structural factors. Findings will inform the development and validation of a national Maternal Needs Assessment Tool to guide integrated maternal and child health programs.

## 1. Introduction

Despite having the largest healthcare expenditure globally (approximately USD 5 trillion in 2023), the United States has the greatest maternal (18.6 deaths/100,000 women) and infant (5.61 deaths/1000 live births) mortality rates among all high-income countries [1,2,3]. Ensuring the wellbeing of mothers and young children is both a public health and economic imperative, and it depends largely on adequate nutrition and care for mothers and their offspring [4,5,6,7]. Optimal infant and young child feeding (IYCF) practices are critical for survival, growth, cognitive development, and overall health within the first 1000 days of life [8]. Exclusive breastfeeding for the first six months, followed by diverse and nourishing complementary foods, with continued breastfeeding through 12 or 24 months, is recommended [9,10]. Yet, in 2022, only one in four U.S. infants under six months was exclusively breastfed, and fewer than 40% received any breastfeeding at 12 months [11].

Among 1.5 million U.S. women in the Pregnancy Risk Assessment Monitoring System, common reasons for early breastfeeding cessation (<10 weeks) included perceived low milk production (57.6%), latching difficulties (38.7%), insufficient milk supply (38.5%), nipple pain (25.5%), infant weight concerns (17.2%), heavy household workload (17.2%), and resuming work (16.7%) [12]. Additional barriers include limited financial resources and family assistance, along with unsupportive cultural norms, hospital practices, and workplace policies [13,14,15]. Reliable information, resources, and support systems can influence breastfeeding duration and exclusivity [16,17,18].

The American Academy of Pediatrics recommends introducing complementary foods at six months, and no earlier than four, to ensure appropriate gastrointestinal function and motor readiness [19]. However, 16% of U.S. infants, and up to 34% in some states, receive solids before four months [20]. Emerging evidence suggests that introducing complementary foods earlier than four months may predispose children to obesity in later life [21,22]. Thus, efforts to promote appropriate complementary-feeding practices during this stage are crucial.

The postpartum psychosocial health and quality of life of mothers are vital for the wellbeing of the mother–child dyad, household, community, and the broader healthcare system [4,5,23,24]. Financial pressures, including the costs of food, childcare, healthcare, and other household necessities, can heighten maternal stress and directly undermine the ability to provide consistent, high-quality nutrition and care for the infant [25,26]. For instance, in a study of 3500 pregnant and postpartum women, one-quarter had unmet healthcare needs, two-thirds could not afford care, and over half reported general financial stress [27]. Logistical challenges such as limited transportation, childcare, and inadequate social support further affect feeding practices [28].

Social isolation and loneliness, affecting up to 30% and 80% of new parents, respectively, often arise from geographical or emotional distance from family and friends, child rearing conflicts with popular culture or expert advice, or the lack of a supportive peer and professional network [29,30,31]. Together, these factors may further diminish informal safety nets and limit easy access to useful IYCF information. Furthermore, inconsistent or inaccurate advice from various sources can heighten confusion and anxiety, underscoring the need for clear, evidence-based guidance [32]. These persistent gaps in infant feeding and maternal psychosocial support highlight the need for systematic identification of the informational, psychosocial, and structural barriers shaping maternal decision-making.

Recognizing these challenges, the U.S. Surgeon General has urged researchers to document parents’ experiences through parent-centered approaches, to inform interventions that support mental health and caregiving [33]. Numerous tools exist for assessing the needs of parents of preterm infants, children with various illnesses, and those in intensive care units, or prenatal needs, but no existing instrument has been identified in the literature for parental needs assessment in child feeding and care of healthy, full-term infants [34,35,36,37]. Existing tools fail to capture the full spectrum of maternal needs, particularly the intersections of child feeding, mental health, and social support, necessitating a more comprehensive, multidimensional approach. Thus, this formative study presents a novel framework that translates qualitative maternal experiences into quantifiable domains for assessment and intervention, a first step in developing a comprehensive instrument, the Maternal Needs Assessment Tool (MNAT). By identifying actionable maternal priorities, this research will contribute meaningfully to U.S. maternal and child health policy and intervention design within the first 1000 days.

## 2. Methods

### 2.1. Theoretical Framework

**Figure 1:**
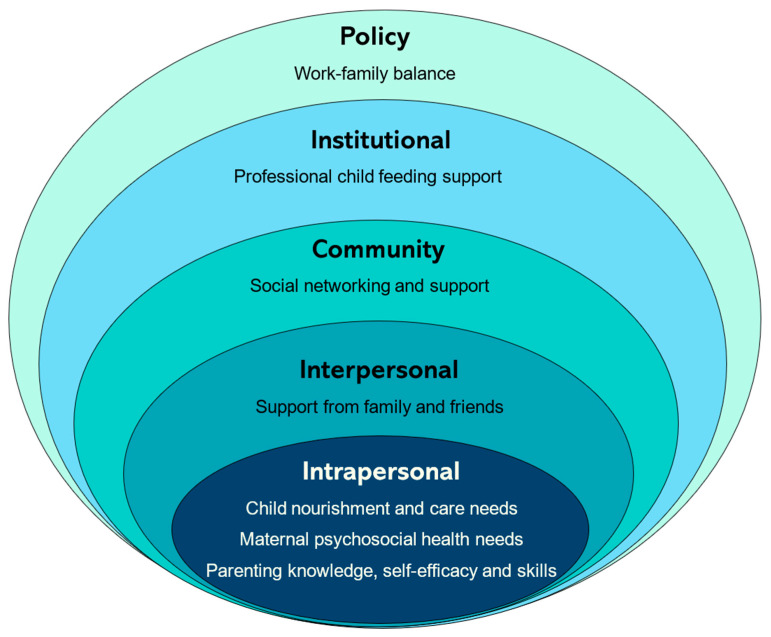
Ecological model of maternal needs for child nourishment and care adapted from McLeroy et al. (1988) [38].

The ecological model of health behavior propounded by McLeroy et al. (1988) was adapted as the framework for this study, as presented in Figure 1 [38]. This framework was chosen to capture how multidimensional, influential factors dynamically interact with maternal feeding and caregiving experiences. This adapted theory centers the child nutrition and care needs of mothers as individuals in need of enhanced knowledge, self-efficacy, and skills. It also explores their interactions with interpersonal influences (needs for support from family and friends), community dynamics (social networking needs), institutional factors (professional IYCF support), and policy frameworks (navigating work–family balance).

### 2.2. Study Design, Setting, and Participants

A qualitative, descriptive design was appropriate for exploring underexamined maternal perspectives and generating data to inform instrument development. Six focus group discussions (FGDs) were conducted via Zoom between March and April 2025. Participants were recruited by in-person distribution of recruitment materials (flyers with QR codes and links to the screening survey) through non-profit and community-based organizations and childcare centers in Buffalo, New York, which work with women with young children. Email recruitment was conducted across the United States (New York, Illinois, Texas, Florida, and other states) via dissemination of digital recruitment materials to organizational listservs by leaders of non-profits and childcare centers, as well as other researchers who work with the target population. Online recruitment was conducted through study-specific social media accounts, with digital flyers posted directly on Facebook, Instagram, LinkedIn, and Nextdoor, as well as shared in both open and closed U.S. parenting groups on these platforms. Recruitment was conducted across multiple states and digital platforms to enhance geographic diversity and relevance to U.S. mothers. Eligibility criteria included English-speaking mothers ≥ 18 years who were first-time parents of a healthy, full-term infant < 2 years, born without complications and free from current illness. Exclusion criteria included preterm birth, birth complications, or child illness. Interested individuals provided online consent before completing an online screening survey which included sociodemographic data. Eligible participants who passed the screening survey provided electronic consent for the study via REDCap. An additional verification step involved a pre-screening Zoom session with cameras on, during which participants answered selected eligibility questions, to confirm that their spontaneous responses matched their survey submissions. Only those whose responses were consistent were scheduled for a focus group discussion on Zoom.

### 2.3. Data Collection

Focus group sessions (FGD; n = 35 participants in 6 groups, 4–8 per group) lasted 60~90 min, moderated by a trained facilitator and attended by a note-taker. FGDs were recorded using Zoom and continued until saturation was achieved. Data from one focus group involving seven fathers were excluded due to minimal engagement; several participants provided limited verbal input, and some appeared to post text responses copied from the Internet into the Zoom chat, rather than contributing to the discussion. Results from the remaining five focus group discussions with 28 mothers are presented. A Focus Group Discussion Guide with open-ended and semi-structured questions was used to explore maternal needs across multiple domains of the socioecological model, related to the wellbeing of the mother–child dyad.

The needs identified during FGDs were compiled into a Likert-type Maternal Needs Assessment Ranking Questionnaire (MNARQ), which was subsequently distributed to all participants for prioritization. A total of 22 of the 28 participants completed the questionnaire. The MNARQ listed maternal needs across 10 domains aligned with FGD themes, broadly covering infant feeding and health, maternal psychosocial wellbeing, and structural support. For each item, participants first indicated whether the need was relevant to them (“yes” or “no”). Participants who responded “yes” were then asked to rank the need on a 5-point scale ranging from 1 (least important) to 5 (most important), allowing quantification of the relative importance of identified needs.

### 2.4. Data Analyses

All Zoom-recorded sessions were transcribed verbatim and anonymized before analysis. Two trained coders independently performed thematic analysis in Atlas.ti (version 25), ATLAS.ti Scientific Software Development, Berlin, Germany [39], beginning with inductive open coding to generate preliminary codes from the data. A consensus-based codebook was then developed. Individual codes (e.g., child-related breastfeeding issues) were consolidated into broader thematic categories (e.g., breastfeeding challenges). This codebook was applied in a second coding cycle to refine, merge, and finalize themes across transcripts. Data saturation was considered achieved when additional data collection or coding no longer produced new themes or insights. Inter-coder reliability was ensured through independent coding, codebook alignment, and consensus resolution by the principal investigator. Axial coding identified relationships among themes and organized them around the domains of the adapted socioecological model. MNARQ data were analyzed descriptively in IBM SPSS Statistics (version 30.0), Armonk, NY, USA [40]. To summarize the relative priority of each need, weighted mean ranks (WMRs) and their standard deviations were computed [41]. This was achieved by multiplying each rank value (1–5) for a given need by the number of participants who assigned that rank to that specific need, summing these products, and then dividing by the total number of responses for that need [42]. Overall mean ranks for each domain were calculated by averaging the weighted mean ranks of all items within that domain, providing a summary measure of the relative importance of each category of needs.

### 2.5. Ethical Considerations

This study adhered to the principles outlined in the Declaration of Helsinki. All research procedures involving human participants received approval (STUDY00009047) by the Institutional Review Board of the University at Buffalo, New York, under expedited review for exempt status. Written informed consent was obtained from all participants.

## 3. Results

The sociodemographic characteristics of participants are presented in Supplementary Table S1. Almost three-quarters of the mothers were <35 years of age, half had completed a graduate degree, and nearly half of the mothers reported being in the low-income group. When asked how long their household could maintain their current standard of living if income were lost, 42.9% reported sustainability for more than 12 months. More than one-third of the children were 6–11.9 months old. The distribution by child sex was equal. The majority of participants lived in smaller households (<three members), while about one-third reported larger sizes.

### 3.1. Study Themes

Seven themes emerged from the focus group discussions, encompassing needs related to (A) infant and young child feeding and care; (B) maternal psychosocial health; (C) parenting knowledge, self-efficacy, and skills; (D) support from family and friends; (E) social norms and networking; (F) support from health and childcare systems and personnel; and (G) work-related policies. These themes were further organized into 10 domains that informed the structure of the Maternal Needs Assessment Ranking Questionnaire (MNARQ) and guided subsequent quantitative prioritization of maternal needs. Table 1 summarizes these needs at different levels of the socioecological framework.

### 3.2. Theme 1: Infant and Young Child Feeding and Care Needs

Needs related to infant and young child feeding and care were organized into four domains: breastfeeding, formula feeding, complementary feeding, and child development, care, and health.

Breastfeeding: While participants recognized the importance of breast milk to infants and had generally approached childbirth with the intention and preparedness to breastfeed, their breastfeeding experiences were fraught with numerous struggles. The highest-ranked breastfeeding needs involved child-related physiological or health barriers, including latching difficulties, conditions such as jaundice, disabilities like tongue tie, aversion to breast milk, and easy distractibility. Maternal challenges, particularly concerns about milk supply and adequacy, were ranked next in importance, often accompanied by feelings of self-doubt or over-pumping of breast milk. One important need documented was for access to high-quality, reliable information on breastfeeding. An unexpected finding was one participant’s difficulty finding accessible, evidence-based information supporting AAP’s two-year breastfeeding recommendation. Her frustration was compounded when her pediatrician dismissed the guideline as relevant only to developing countries.

*JE: So, um. Being a pediatrician, I thought I knew how to do it. But being a first-time mom, I clearly did not know what I was doing. And so had a lot of pain and damage and latch issues early on.*

*VR: So I had a goal to breastfeed my son before he was born, and he also had a tongue tie. It was very frustrating at first, but I kept trying, and I would talk to his pediatrician, and we had a lactation consultant at his pediatrician’s office. I remember I gave him just 2 ounces of formula, and I felt so bad, because my goal was really to breastfeed him. They introduced me to a nipple shield which helped a lot, so we were able to breastfeed without him having a procedure. I pump when I’m at work. He’s almost 10 and a half months, and my supply has been dipping recently, so it’s been more feelings of stress to make it to our goal of a year.*

*AR: Since the AAP raised the breastfeeding guideline to two years, I tried to find high-quality information to justify continuing, but it was really hard. I read a lot online, even doom-scrolled, and talked to my pediatrician and a lactation consultant. My doctor eventually said the two-year recommendation was based on WHO guidance for global populations, not necessarily ours, since we have access to other nutritious foods.*

Formula feeding: The two topmost ranked formula-related concerns were allergies and conflicting guidance/confusion in formula selection. Common challenges experienced by study participants included lack of affordability and access to infant formulas and lack of clear expert guidance on how to determine the best infant formula. Some mothers also reported unintended early introduction of infant formula either due to pressure from health professionals or the infant’s breast milk aversion or allergies. Also, participants expressed discomfort with the numerous hurdles they had to navigate in order to receive alternative brands of infant formula in situations of infant rejection or reaction to formula. These include booking doctor’s appointments, completing paperwork to document the problem with the current formula, presenting the paperwork to WIC, and waiting for approval.

*EC: And also you should look into the formula feeding guides. They should try to give information on what to choose, like the right formula, and then how to prepare the bottle for the child and also try to manage the formula feeding.*

*AF: Okay, so when we were in the hospital, they gave us Enfamil. And he (my son) rejected Enfamil. He’s a very picky baby so Enfamil, he could not take and we had to switch to Similac. And Similac Sensitive was the only one that he would take. We would try you know different ones, but Similac Sensitive was the only one he wanted. And so when it came to the WIC, in order for me to get the only formula that he can drink, which is Similac, I had to get a doctor’s note to prove to them like, hey, the baby is only drinking this and this is what we can only give him. And so if I didn’t have that doctor’s note, they weren’t going to be able to supply me with that specific formula. I would have had to have 20 plus dollars out of pocket every time for each can. And it’s only like 12 ounces in a can of powder. And so I’m like, $20 for 12 ounces, that’s gone within three, four days. And so imagine having to buy four or five cans every week and that gets pretty expensive.*

Complementary feeding: The highest-ranked complementary-feeding priorities were ensuring the provision of safe foods to prevent choking, managing food allergies, and modeling healthy eating behaviors. As infants transitioned to solid foods, mothers emphasized the need for clear expert guidance on the timing of the introduction of complementary foods, infant-appropriate food types, textures, and portion sizes, and practical skills for preparing healthy meals that align with both nutritional guidelines and family routines. They also struggled with balancing their personal child feeding paradigms with popular feeding approaches, such as baby-led weaning. Finally, mothers expressed a need for guidance on how to navigate frequent stressors including picky eating, gagging on solids, food allergies, and rejection of new foods.

*CC: I do wish, like my doctor gave more specifics about the food, like, what foods to try first, and when and what to do, like what to look for allergies. That sort of thing. Uh, or at least point me to resources. And there’s a million things on the Internet, but what are the most reliable sources? Or point me towards like the better like things to read in terms of like being valid or researched.*

*JE: Making sure he has a healthy diet is a top concern. I am not always the most healthy diet person. I’m definitely a chicken tenders and French fries girl, which I blame my mom for, but that’s okay. And so, of course, that’s what we eat a lot at home. I’m not the biggest veggie eater. And so I’m just trying to be very mindful of what I feed him and trying to not you know just give him chicken nuggets and French fries but expand all of those things.*

Child development, care, and health: The most highly prioritized needs related to child development, care, and health were ensuring optimal infant growth, fostering early parent–child communication, and engaging the child through play. Mothers also highlighted a desire for training to help in recognition of physical and cognitive developmental milestones, interpreting infant cues, establishing healthy sleep schedules, promoting appropriate socioemotional development, and encouraging active lifestyles for the child’s long-term wellbeing. Mothers whose children experienced delayed milestones described significant anxiety and stress, expressing a desire for “baby boot camps” to help children catch up by fostering parent peer connections, creating opportunities for child interaction, and providing professional guidance to support milestone achievement.

*PW: So child development also is very important. And when to know that your child is developing in the right way, or when you need to ask, or when you need to be concerned in case of anything. Because I’ve not seen, like, so far, any information about that. I just rely on what I Google online, because sometimes you might have concerns. But you just have to Google and check, but you don’t know even if that’s a reliable source that gives you that information. I’m just nervous, because I want to know if she’s meeting her milestones at the specific age, if she’s having the right weight, because sometimes you go, they tell you ‘Oh, she’s not meeting her milestones’, so it’s very stressful for a mother. So that is what I usually worry about meeting milestones and just being healthy because it can be stressful when they are not healthy, and especially if you don’t know what the problem is.*

*EF: What a mother is always afraid of is when you give birth to your child at that early stage, then you lose your child. That is the most painful thing.*

### 3.3. Theme 2: Maternal Psychosocial Needs

While a few mothers described experiencing clinically diagnosed postpartum depression, almost all participants reported feeling overwhelmingly fatigued and burdened by the relentless pace of caregiving responsibilities, the highest-ranked psychosocial need. These feelings were often compounded by sleep deprivation, feelings of perceived inadequacy in parenting, and mom/separation guilt on return to work or sending the child to daycare. To deal with these psychosocial concerns, mothers articulated a need for support and resources to help prioritize their own happiness and mental wellbeing, the second-highest-ranked need, viewed as essential to their ability to nurture their children.

*DC: But, like, you know, when you finally have your baby, and you know, this is actually reality. No one tells you really, that postpartum depression hits you like a train. You just don’t feel like yourself, and then you’re left wondering, you know, like, is this normal? Will this ever, you know, get better? And thankfully, you know they do have treatment, but I think something that would be better, or at least for me, I wish there was some kind of information that I was given about postpartum depression and like options sooner before I was like 2 months into it and miserably, you reached out to the doctor and got help.*

*CH: I think I did have more of a postpartum depression period where I felt just different, and like um. It was really hard to enjoy that time with the baby when my hormones were just out of whack! After having my baby my own wellbeing kind of took a backseat. There were days I felt isolated and emotionally drained, when I didn’t feel like I had the space or time to prioritize self-care. I think a lot of moms go through that, but don’t always talk about it.*

### 3.4. Theme 3: Parenting Knowledge, Self-Efficacy, and Skills

The highest-ranked parenting knowledge and skill need was access to consistent, trustworthy health information. Study participants underscored the importance of parenting training but highlighted significant gaps in access to free or affordable pre- and postpartum education, particularly on infant and young child feeding, dietary guidelines, and general health knowledge. Many mothers shared a desire for open and honest peer-to-peer knowledge sharing with other mothers in similar contexts. They also highlighted a need for guidance on where to find reliable parenting information and how to acquire hands-on parenting skills, including time management, child growth charting, swaddling and soothing a baby, household work burden/meal preparation, and evaluating their own success as caregivers. These mothers stated a preference for flexible training delivery through multiple formats—in person, online platforms, apps, newsletters, podcasts, workshops, and hands-on sessions.

*EC: I would be interested in like child development courses and some parenting skills. I would prefer some online resources like there should be parenting books and online parenting courses. If possible, there should be online parenting apps which can actually help some new parents.*

*C: I did some training before the birth of my child. There also was a Lactation Center nearby, called the Care Connection, that did the infant CPR and a breastfeeding class. So those are all great in person things where you could talk to other people. You actually had like a doll, or like a dummy, you know, to play with, uh to practice with. You can practice like swaddling a baby. All of that, I think, is really helpful. But again, like the hands on, once you have the child, you still need another refresh, or a person that’s right there with you in the hospital, too, to support you, so I think both are important for different reasons. But um, in person trainings and interacting with other moms is so helpful, so they can share their experiences.*

### 3.5. Theme 4: Support from Family and Friends

The most highly prioritized interpersonal needs were addressing trust issues with external caregivers and improving proximity to family support. Participants emphasized the importance of family and social support in managing early parenting challenges but described these networks as often absent, reduced, or strained by conflict. Many highlighted the lack of a “village” to rely on, leading to feelings of isolation. Even mothers with nearby relatives or friends reported limited assistance, as others were preoccupied with their own families. While some participants felt adequately supported, others noted minimal hands-on help, particularly when spouses worked long hours or their family lived far away. Interpersonal conflicts within the home, unrealistic spousal/cultural expectations surrounding parenting roles, and varying levels of spousal support added further strain.

*LB: I think I’ve lacked a lot of support. My partner works um he works an hour and a half away from where we live. So he commutes about three hours a day, which is a lot. So he’s gone from like 5:30 in the morning until 8:30 at night. So I’m with the baby all day, which I love. And I love that I have this time. I’m still on leave, which is amazing up until a year. So I feel really fortunate that I have this time with him, but I definitely feel like I don’t have a lot of support. And my parents have sadly passed. So my sisters will come down occasionally and the other side of my baby’s family is in the Midwest. So I don’t feel like I have that village to help. So a lot of it has fallen on me. And my partner is good when he has time off. He helps with like meal preparation, cleaning, and he’s really amazing about all of that stuff but during the week, I definitely take on those roles um and so It can kind of get to me where I definitely require time to myself to help relax my mind and to process things. And so that’s been a challenge for me not having that time. So yeah, it does fall a lot on me. And I definitely think like You know, they say it takes a village. And I think when you don’t have that village, your mental health does suffer a little bit. Like we all need support.*

*LB: So that’s been a struggle just because, like my partner is a little bit more lax about things. So getting him to be on the same page as me and also dealing with like the in-laws and them having their opinions around like oh you should be adding rice cereal to his milk. It’ll make him sleep better. And I’m like, I don’t like the arsenic levels in the rice cereal. You know, like there’s just a lot of things that people did 50 years ago that they don’t do now. It’s hard to talk to people about that and have them understand where you’re coming from without them finding it disrespectful. So those conversations were challenging. But also just having to like be firm in my boundaries and like my child’s health is the most important thing. And so I’ve had to like put my foot down about some things and then I worry like, oh, if I leave him alone with them, are they going to just do it anyways, which luckily they live in Chicago, while I’m on the West Coast. So they’re not close by. So that’s a nice boundary in itself too. That makes me worry less.*

*LC: mm, but now, interestingly enough, our nanny, she’s amazing, but what we are struggling with, with her is I think she’s scared to give my daughter, anything other than a pureed food. And I’m not really adhering to pureed or baby led weaning, it’s kind of like whatever we’re eating, just give it to her. I don’t really care and just monitor her. But she’s absolutely terrified to give her anything that’s not pureed and like a stage 1 baby food kind of thing, so I’m struggling with like, how do we educate the nanny of like what is appropriate? Are we holding back development, you know, in terms of her trying different textures because the nanny that’s with her 50% of the time, won’t give her what I feel like is appropriate. Umm we did have to have a conversation with the nanny about like, you know if she’s more cranky or more hungry, here are other things to try because the nanny at one point was using like way more milk, to the point we were digging into my freezer supply during the day umm but not the days where the baby was only with my husband, it was only when the nanny was there we were having to dip into the freezer because she was feeding her so much milk. And so we kinda had to do some education of like, okay here’s what we’re gonna do instead of tanking mom’s milk supply because we’re trying to quiet the baby who won’t take a pacifier. And so, we’ve had some of the same struggles but it’s been a journey for sure.*

### 3.6. Theme 5: Social Norms, Networking, and Support in the Community

The most important community-level need identified by participants was access to peer support groups and community networks to share childcare responsibilities, reduce isolation, and validate mothers’ experiences. Some mothers highlighted the value of rotating childcare arrangements within their community, which allowed some members to provide care while others rested or engaged in stress-relieving activities.

For immigrant mothers in the study population, these pressures were compounded by divergence between acceptable child rearing practices in the United States and those in their home cultures. Additionally, some mothers struggled with conflicts between personal preferences in child rearing and the recommendations from medical practitioners and the prevalent practices in their communities. For instance, some mothers voiced concerns about the pressure from some community/family members and health workers to practice exclusive breastfeeding, when they would rather not. Mothers from the Black community described the absence of breastfeeding role models among immediate family members and friends. These mothers experienced social stigmatization arising from breast sexualization, isolation and undue pressure to practice early breastfeeding cessation.

One unique finding is that a participant expressed a deep concern that the conflicting and hostile political and ideological views of their immediate circle of neighbors and colleagues would negatively affect the psychosocial development of their child, highlighting a need for guidance on how to navigate these challenges. Collectively, these narratives denote a strong need for community networks, peer support groups, and other platforms to share childcare burdens, reduce isolation, and validate mothers’ experiences. The desire for community/friend support groups to help with childcare emerged as the most important community need.

*PW: For breastfeeding, like in my culture, you’re not supposed to breastfeed like in front of people. You have to go somewhere, or you have to cover yourself, which is something I had not also expected or seen before, because when you talk to other people they’d be like, ‘Oh, when the child is hungry, just feed them’. But then there’s a cultural difference. You can’t just feed the baby anywhere. You have to look for a secure place, then sit down and maybe cover yourself, then feed the baby there.*

*CC: And there was actually this pressure to exclusively breastfeed. But it wasn’t working for us. Well, even though it was the right decision that I made, I felt a lot of judging around it.*

*AR: I think raising her in a society or bubble, I guess, of like good people is very important to me. I’m very surrounded by the federal government and government employees, politics. And kind of the hatred directed at us and it’s really, really hard for me to think about how to raise her in a society that has the values that I want to impart and avoid the values that I don’t want. And when it seems to me like we’re so surrounded by views that I find abhorrent. It scares me about like how to not raise her that way.*

### 3.7. Theme 6: Support from Health and Childcare Systems and Personnel

The most frequently cited institutional need was access to professional medical, feeding, and childcare guidance from health providers, including lactation specialists, nurses, and pediatricians. One mother described irreversible setbacks to establishing breastfeeding after delivery due to inconsistent nurse/lactation support and being overlooked by hospital staff assisting other mothers.

Mothers also reported other health system gaps: limited or nonexistent postnatal home healthcare, insufficient pediatric visits, inadequate lactation support, conflicting formula-feeding guidance, and few resources for student mothers. These challenges were compounded by transportation barriers and limited virtual appointment options. A few mothers in one Midwestern state described benefiting from a state-funded Healthy Beginnings program that offered regular home visits for early detection and treatment of mother–child health concerns in the first 1000 days. Others, lacking comparable programs in their communities, expressed a strong need for similar free or insurance-covered services.

Some mothers also faced daycare policies that restricted bottle feeding despite a child’s needs or refused expressed breast milk after certain ages, undermining breastfeeding goals of up to two years. Additional barriers included requests for larger breast milk volumes to match formula-fed quantities and concerns about illness transmission in daycare settings.

Collectively, these experiences underscore the need for accessible, affordable, and consistent parent- and child-centered support across healthcare and childcare systems, as well as policy reforms that protect breastfeeding and promote maternal and child wellbeing.

*OA: So for me, the hospital I was registered at had a special program called the Healthy Beginnings where they have, like, a home nurse who comes to visit you before and during pregnancy and after birth, and even right after the baby is born. She still visits till now. So she made available so many resources, information, and all of those things, and I remember, after I got back from the hospital, in one of our visits, she discovered that my blood pressure was really high, and she sent me back to the hospital. And with such resources. It saved my life at least, and I know that for many others who are in the network the different nurses at the Healthy Beginnings program help the patients with food related information, information in terms of how to take care of the baby. They provide pamphlets that contain information on what to expect postpartum in terms of milestone for the kids, and all those things.*

*JE: And I think one of the issues that I ran into with breastfeeding was when he moved up in classrooms at daycare, they were pretty adamant that like “breast bottles and breast milk was done.” And he literally turned one and moved up like three days later. And I was like, well, I can get down with taking the bottles away because like I said, I’m in healthcare, so I wanted to get rid of the bottle, but I had pumped like 400 ounces of breast milk. And I was like, I want him to get this breast milk. And so it was a little bit of a challenge with the daycare.*

### 3.8. Theme 7: Work-Related and Social Policies

Balancing motherhood with employment emerged as the top work-related policy concern. Many mothers described the strain of serving as both financial providers and primary caregivers, compounded by inadequate maternity, family, and sick leave. These pressures led to stress, guilt, and, for some, the need to reduce work hours or leave employment altogether—intensifying psychological distress and financial strain amid high childcare, formula, and household costs.

Mothers also reported added burdens from employers, such as covering colleagues’ shifts. While a few felt supported at work, others faced job insecurity and lacked family-friendly policies, including lactation rooms or quiet spaces. These conditions hinder mothers’ ability to care for themselves and their infants, underscoring the need for stronger system-wide supports for working mothers.

Regarding food assistance policies and programs, mothers also noted the need for more family-friendly resources, including child-appropriate food pantries for families facing food insecurity.

*EF: The house chores were stressful, then the household meal preparation too. Because I wouldn’t have the time to prepare meals for my baby and all of that, then also check up on myself. It was always so tiring. When my baby falls asleep, that’s the only time I can squeeze just to manage to prepare something for my own self and also try to prepare food that when my spouse comes back he can go on with that. So the challenge was always too much on me.*

*DC: And then I also found it hard to return to work after having my baby. I just had no motivation to, you know, even attempt to go back. And even though I was working remotely, I still didn’t have the energy or desire to go back to work. And then, of course, the work–family balance as well—once I finally started going back to work, it was also difficult because it seems like with your baby, it’s never done. There’s always something they need. It’s the never-ending task of being a mom, I guess. And I love every second of it, don’t get me wrong, but it does make it harder to find that balance between work, taking care of the baby, and quality time with your family.*

*Yeah, when it came to the maternity leave, it was really limited. I only had a short time, and even that wasn’t fully paid, which added a lot of financial stress. It felt like I was being pushed to recover and return to work before I was emotionally ready. I also struggled with sick leave—it wasn’t always easy to take time off, and I constantly worried about job security. There just isn’t enough time for working parents.*

**Figure 2:**
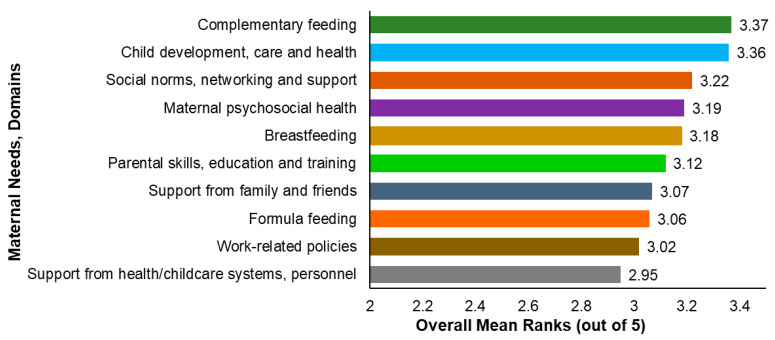
Overall mean ranking of maternal needs across domains.

In Figure 2, the overall mean ranking of maternal needs across the identified domains is presented. The highest overall average rankings were observed in child development, care, and health, complementary feeding, social norms, networking and support, maternal mental health, and breastfeeding. In contrast, structural and logistic barriers seemed comparatively less pressing, as reflected in lower overall rankings for support from medical and childcare systems and work-related policies. These patterns suggest that interventions to support mothers should prioritize both infant care and maternal wellbeing, while also considering the broader structural barriers which, although ranked lower, may compound maternal child rearing efforts.

## 4. Discussion

This formative study integrates qualitative insights from focus group discussions with quantitative prioritization to elucidate the multidimensional needs for child nourishment, care, and support among U.S. mothers of healthy term infants < 2 years. This mixed-methods design marks a methodological advancement, because it captures the richness of maternal narratives and systematically translates the lived experiences of mothers into measurable domains of need. Thus, it lays the foundation for the development of the Maternal Needs Assessment Tool (MNAT). Unlike existing needs assessment instruments that focus narrowly on clinical or psychosocial dimensions, such as the NICU Family Needs Inventory or the Psychosocial Needs Self-Assessment Scale, the proposed MNAT integrates physiological, behavioral, structural, informational, and sociocultural factors to provide a comprehensive framework for maternal wellbeing [43,44].

To our knowledge, this is the first study to systematically identify and quantify the needs of mothers of healthy children using a socioecological lens. The findings present a systems-oriented conceptualization of maternal wellbeing encompassing seven themes across ten domains, spanning child feeding and care, maternal psychosocial health, and structural support. The results demonstrate how multiple socioecological factors (intrapersonal, interpersonal, institutional, community, and policy) interact dynamically to influence maternal and infant health during the first 1000 days of life. For instance, intrapersonal stressors such as feeding challenges and fatigue were intensified by gaps in social and professional support, institutional barriers to care, and policy constraints on work–family balance.

### 4.1. Intrapersonal Determinants

The intrapersonal needs of mothers were organized into three themes, viz. IYCF and care needs (breastfeeding, formula feeding, complementary feeding, and child development domains); maternal psychosocial needs; and parenting knowledge and skills. The high salience of child-related difficulties and mothers’ perceived breast milk insufficiency further reaffirms that breastfeeding success is a dynamic dyadic process dependent on the mutual biological and emotional regulation of mother and child [45,46,47]. While some mothers responded with self-doubt and anxiety to this insufficiency, others responded by over-pumping, which is a well-intentioned but paradoxical, compensatory strategy [48,49]. Both responses may exacerbate maternal stress in a negative feedback loop, possibly culminating in a downward spiral of worsening breastfeeding difficulties or other adverse sequelae [50]. This highlights the need for integrated interventions that pair emotional regulation and self-efficacy training with practical lactation support.

Beyond breastfeeding, mothers’ formula-feeding experiences revealed additional informational and structural gaps. The most important formula-feeding concerns of mothers were child-related allergies/negative reactions necessitating formula switching. Maternal reports of difficulties navigating WIC formula substitution processes in this study are consistent with prior findings of inconsistent access to allergy-safe foods through WIC/SNAP programs, yet they contrast with WIC staff perceptions that formula substitutions are readily implemented. Although anecdotal evidence of barriers to infant formula substitution exists, systematic empirical research on this issue remains scarce, underscoring the need for further scientific investigation [51,52]. Additionally, confusion in infant formula selection reported in the current research may be fueled by market-driven exaggerated and ambiguous health claims, coupled with inconsistent professional advice. These challenges may erode confidence and distort feeding decisions [53,54].

Complementary feeding is a critical yet challenging period of transition. Safety concerns, particularly fear of choking, and food allergies and picky eating, which received the highest ranks in the present study, may promote delayed introduction of complementary foods [55,56]. Although evidence that early feeding struggles may predict later dietary selectivity is inconclusive, the acquisition of food preferences appears to be time-sensitive, and picky eating may compromise nutrient intakes and modulate long-term developmental and health status [57].

The foremost child development and care need was ensuring optimal infant growth and overall wellbeing, followed by fostering early parent–child communication and psychosocial development. Although national polls identify psychosocial development as parents’ top concern, our findings contrast with this trend, showing greater emphasis on physical rather than socioemotional growth. This discrepancy is worth exploring, as physical growth concerns may be more intense for parents of infants than the general parent population [58,59,60]. Furthermore, mothers whose children experienced developmental delays described significant anxiety and a desire for structured, catch-up programs like “baby boot camps”. Additionally, maternal training in recognizing infant cues and establishing appropriate sleep schedules, although ranked lower, may help infants learn to differentiate hunger from other needs, prolong and consolidate nighttime sleep, and improve self-soothing [61,62].

Concerning maternal mental health, our findings show that participants rated feeling overwhelmed, fatigued, and burdened by parenting responsibilities the highest, consistent with national patterns [32]. Participants voiced a strong desire for resources to help them prioritize their own happiness and mental wellbeing. Overall, these findings point to the need for practical, evidence-based strategies that strengthen maternal confidence, improve access to reliable guidance, and address the structural barriers that hinder optimal infant feeding and care.

### 4.2. Interpersonal and Community Dynamics

The top concerns across the family and friends support and social norms and networking domains included trust issues with external caregivers and lack of proximity to family. Mothers lamented the erosion of the “village” that traditionally supported new parents. Despite logistical challenges, participants valued peer connection and collective childcare arrangements as sources of emotional relief. These findings support expanding community and digital peer support initiatives, which have been shown to improve maternal confidence and reduce depressive symptoms [63,64].

### 4.3. Institutional and Workplace Barriers

The most pressing structural needs included access to professional feeding and childcare guidance and support for balancing motherhood, work, and family responsibilities. Participants expressed frustration with inconsistent healthcare engagement, limited postpartum follow-up, and inadequate lactation and pediatric support. Those who benefited from comprehensive home-based programs like *Healthy Beginnings* reported markedly better outcomes, underscoring the value of intersectoral, preventive, and family-centered services rather than fragmented and reactive models of care [65]. Scaling such evidence-based models could close persistent service gaps.

Mothers also described an “information paradox”, an abundance of online content but a scarcity of reliable guidance. While digital platforms offered convenience and peer connection, conflicting information often fueled confusion and anxiety [66]. This highlights the need for curated, evidence-based digital resources that combine clinical accuracy with peer relatability. Public health agencies could collaborate with trusted digital innovators to develop interactive, verified education platforms. Finally, expanding paid family leave, ensuring workplace lactation accommodations, and investing in affordable childcare would address the structural roots of maternal exhaustion and inequity.

### 4.4. Cross-Cutting Issues

Although many of the needs identified in this study have been described individually in the prior literature, the novelty of this work lies in integrating these components into a unified, theory-informed framework. This synthesis will directly inform the development of the Maternal Needs Assessment Tool (MNAT), providing a structured and measurable approach to assessing maternal needs that has not previously existed.

A critical yet underexamined finding concerns how race, culture, and socioeconomic context intersect to shape caregiving realities. Black and immigrant mothers described unique sociocultural pressures, including breastfeeding stigma, conflicting motherhood ideals and barriers to culturally sensitive professional guidance. These findings align with national data showing persistent inequities in breastfeeding and postpartum support among racially minoritized and immigrant populations [67]. To address these gaps, culturally responsive models of care that integrate tailored lactation counseling and community role models are needed to ensure success across the sociodemographic continuum.

Moreover, several mothers reported feeling dismissed or stereotyped by healthcare providers, experiences that diminished confidence and trust. This aligns with evidence linking relational disrespect and implicit bias in perinatal care to reduced adherence and satisfaction [68,69]. Embedding relational competence which embodies listening, empathy, and shared decision-making into health and childcare systems, along with trauma-responsive and bias-prevention training, could strengthen trust and engagement.

Mothers commonly reported fatigue, guilt, and difficulty balancing work, childcare, and household demands, a persistent challenge among U.S. working mothers exacerbated by limited childcare access and inadequate parental leave [70]. Reducing this invisible burden of unpaid labor requires expanding affordable childcare, promoting shared domestic responsibilities, and adopting flexible work policies to support maternal and child wellbeing.

Finally, an emergent opportunity lies in rebuilding the “village” mothers long for through both digital and community innovations. Virtual peer networks, local childcare cooperatives, and parent-led advocacy initiatives could engineer informal systems of resilience and shared care [71]. Supporting these grassroots solutions through public–community partnerships and sustainable funding can enhance reach and foster collective wellbeing.

## 5. Limitations

Several limitations of this study should be acknowledged. The small sample size (*n* = 28 for FGDs, *n* = 22 for the MNARQ) limits the generalizability of findings to broader populations. Participants were predominantly English-speaking, educated U.S. mothers, so experiences of rural or culturally diverse populations may differ significantly. As a self-reported and cross-sectional study, the data reflect perceptions and priorities at a single time point, without accounting for changes as infants grow or mothers’ circumstances evolve. Because of this design, the findings capture reported needs rather than causal relationships, and no causal inferences can be drawn. Inconsistencies between the reported and observed demographics of participants skewed the sample and led to the addition of a pre-screening step for verification. Furthermore, the MNARQ, derived from FGD themes, has not yet undergone psychometric validation and should be interpreted as a formative tool guiding the development of the comprehensive MNAT to be validated in the next research phase. While mean rankings and weighted scores provide descriptive insight, they cannot establish causation or fully capture interdependencies among domains. Moreover, weighting assumes equal interpretive understanding of ranking scales across participants, which may not always hold. Coding was performed by two trained analysts with consensus adjudication. Future work will expand sample diversity and validate the MNAT through exploratory and confirmatory factor analyses.

## 6. Conclusions

In sum, the findings of this study emphasize that the needs of mothers transcend discrete categories of personal child feeding or health domains. Rather, they exist within a web of intersecting interpersonal, social, cultural, and systemic forces. Intersectionality, relational respect, informational clarity, time equity, and structural integration emerge as cross-cutting priorities that future research, policy, and practice must address. Recognizing and embedding these principles into maternal and child health initiatives will be pivotal for advancing empowerment and sustainable wellbeing among U.S. mothers and their young children. This study thus contributes critical, context-specific insights for developing a Maternal Needs Assessment Tool that captures mothers’ multifaceted priorities. Such an instrument could guide public health programming, policy development, and individualized care planning, ultimately advancing maternal and child health equity in the United States.

## Figures and Tables

**Table 1:** Maternal needs for child nourishment, care, and support across socioecological levels.

Socioecological Level	Emerging Themes	Maternal Needs	Weighted Mean Rank (SD)	N
Intrapersonal	Infant and young child feeding and care needs	Breastfeeding		
		Mother-related breastfeeding challenges		
		- Concerns about breast milk supply and adequacy, leading to over-pumping, and self-doubt	3.47 (1.25)	14
		- Maternal health and physiological barriers (hormonal imbalances, cracked nipples/mastitis)	3.33 (1.40)	15
		- Challenges with breast milk expression (aversion to pumping, exclusive pumping, breast pump attachment)	3.20 (1.32)	10
		- Expert breastfeeding guidance (quantities, duration, benefits and scientific basis for AAP recommendations on exclusive breastfeeding, breastfeeding goals and expectations)	3.07 (1.25)	15
		- Stress and burden of continuous breastfeeding	3.17 (1.19)	12
		- Aversion to breast milk expression	3.00 (1.41)	9
		- Challenges with establishing supply after supplementing with infant formula	2.50 (1.08)	10
		Child-related breastfeeding issues:		
		- Latching, ill health, disability, breast milk aversion, distractibility	3.71 (1.26)	17
		Formula feeding		
		Mother-related formula-feeding challenges		
		- Access and affordability barriers	2.75 (0.71)	8
		- Conflicting guidance and confusion in formula selection	3.25 (1.04)	8
		- Unintended early introduction of formula	2.89 (0.78)	9
		Child-related issues		
		- Infant rejection of formula and difficulty finding acceptable options	3.14 (0.86)	14
		- Allergies or negative reaction, with no options except breastfeeding	3.27 (1.10)	11
		Complementary feeding		
		Mother-related complementary-feeding challenges		
		- Knowledge of right timing, type, amounts and procedures for introducing solids	3.54 (1.13)	13
		- Concern about food texture to prevent choking	3.77 (1.17)	13
		- Skills in food preparation and nutrition (child-appropriate, healthy, household meals/snacks, obesity prevention)	3.37 (1.03)	16
		- Concerns about long-term impacts of convenience foods	3.00 (1.25)	10
		- Modeling appropriate eating behavior	3.55 (1.13)	11
		- Conflict with popular infant feeding principles (e.g., baby-led weaning)	2.90 (1.29)	10
		Child-related issues		
		- Navigating food allergies	3.55 (1.13)	11
		- Rejection of complementary food, gagging, and picky eating	3.25 (1.49)	8
		Child development, care, and health		
		Ensuring optimal infant growth and overall wellbeing	3.70 (0.95)	10
		Recognizing infant cues (hunger, thirst, satiety) and developmental milestones	3.20 (0.79)	10
		Preventing disease conditions, including those from childcare centers	3.27 (1.03)	15
		Establishing an appropriate infant sleep schedule	3.30 (0.82)	10
		Promoting psychosocial development and emotional intelligence	3.58 (1.31)	12
		Preventing infant mortality	3.42 (1.17)	12
		Fostering early parent–child communication and entertaining the child	3.67 (1.07)	12
		Preventing sedentary lifestyles	2.75 (1.29)	12
	Maternal psychosocial needs	Prioritizing happiness and mental wellbeing	3.42 (1.24)	12
		Feeling overwhelmed, fatigued, and burdened by parenting responsibilities	3.50 (1.32)	16
		Sleep disturbances	3.18 (1.24)	17
		Specific individual needs for support	3.22 (1.39)	9
		Perceived inadequacy as a parent	3.13 (1.13)	15
		Mental health challenges and postpartum depression	3.21 (1.48)	14
		Separation guilt when child goes to daycare	3.23 (1.42)	13
		Feelings of guilt for returning to work	3.15 (1.14)	13
		Concerns about sending young infants to daycare	3.07 (1.14)	14
		Navigating parental self-care	2.77 (1.24)	13
	Parenting knowledge, self-efficacy and skills	Inadequate access to affordable pre- and postpartum parenting education on - Breastfeeding and complementary feeding, child nutrition, and health knowledge	3.33 (1.16)	13
		Insufficient access to free parenting resources	3.00 (1.32)	9
		Lack of honest peer-to-peer knowledge sharing	3.18 (1.25)	11
		Challenges with identifying reliable information sources	3.43 (1.22)	14
		How to develop parenting skills to overcome challenges	2.92 (1.19)	13
		Learning patience	3.29 (1.33)	14
		How to self-evaluate success in parenting	3.00 (1.35)	12
		Appropriate format and timing for parental training (classes, online, workshops, hybrid, video, hands-on education)	2.64 (1.64)	14
		Time management issues	3.15 (1.14)	13
		Navigating household work burden/meal preparation	3.29 (1.07)	14
Interpersonal needs	Support from family and friends	Need for breastfeeding role models	3.08 (1.38)	12
		Lack of child rearing support	2.50 (1.18)	10
		Conflicts within the home or spousal relationship/partnership	2.82 (1.78)	11
		Cultural expectations of mother’s role and spousal support	3.30 (1.16)	10
		Being the only one who can soothe the infant	2.89 (1.27)	9
		Lack of proximity to family	3.36 (1.15)	14
		Trust issues with external caregivers, including family, nanny, daycare, and others who may have conflicting views on infant feeding	3.55 (1.04)	11
Community-related needs	Social norms, networking, and support	Challenge with meeting general motherhood standards in the community	3.20 (1.14)	10
		Cultural norms around child rearing (mismatch between heritage and surrounding culture)	3.11 (0.93)	9
		Societal breastfeeding stigmatization	3.29 (1.27)	14
		Societal/family/health worker pressure to breastfeed exclusively	2.83 (1.40)	12
		Conflicts between cultural and medical practices	3.22 (1.20)	9
		Maintaining social connections and navigating isolation	3.19 (1.42)	16
		Need for community/friend support group to help with childcare	3.58 (1.24)	12
		Navigating national politics and external influences	3.36 (0.92)	11
Institutional needs	Support from health and childcare systems and personnel	Need for access to medical, professional feeding, and childcare advice	3.45 (1.13)	11
		What to do when professional child feeding and care recommendations fail	3.17 (1.12)	12
		Inadequate postpartum professional care	2.88 (0.99)	8
		Support needs after C-section	2.91 (0.94)	11
		Need for circumcision training	3.20 (1.14)	10
		Insufficient pediatrician appointments	3.00 (1.16)	10
		Insufficient/inconsistent lactation support	2.70 (1.49)	10
		Navigating parenting as a student	3.22 (1.09)	9
		Lack of health institutional support	3.00 (0.71)	9
		Logistical issues with medical appointments (transportation, wait times, lack of virtual options)	3.00 (1.00)	7
		Language barriers with health professionals	3.22 (1.39)	9
		Need for postnatal home healthcare	2.86 (1.22)	7
		Logistics and other forms of support	3.00 (1.31)	8
		Pediatrician dismissed the need to breastfeed for 2 years	2.17 (0.75)	6
		Lack of available and affordable childcare solutions and daycare inflexibility	3.11 (1.45)	9
		Daycare not accepting breast milk when child turns one year	2.63 (1.06)	8
		Daycare demands larger quantities of breast milk to match formula-fed kids	2.73 (1.19)	11
		Limitations on bottle feeding as infant moves up in daycare	2.78 (1.20)	9
Policy frameworks	Work-related policies	Balancing motherhood, work, and family obligations	3.69 (1.08)	16
		Insufficient maternity/family/sick leave and unpredictable health events	2.78 (1.48)	9
		Burdens created by employer and lack of job security	3.22 (0.83)	9
		Need for a family-oriented work environment	3.25 (1.22)	12
		Financial concerns (childcare, formula affordability, early return to work)	2.90 (1.37)	10
		Conflicting financial provider and childcare roles	2.58 (1.31)	12
		Hidden and unpaid labor (home management, child rearing, mental work, etc.)	2.67 (1.00)	9
		Need for child-appropriate, family food pantry for parents	3.09 (0.94)	11

## Data Availability

The raw data supporting the conclusions of this article will be made available by the authors on request.

## Supplementary Materials

The following supporting information can be downloaded at https://www.mdpi.com/article/10.3390/nu17243825/s1, Table S1: Sociodemographic characteristics of mothers.
