# Supplementary material for: “What Do You Need?” Formative Research to Develop a Comprehensive Maternal Needs Assessment Tool for Infant and Young Child Nourishment and Care in the United States
Source: Nutrients. 2025 Dec 6;17(24):3825. doi: 10.3390/nu17243825 (PMC12735840; doi:10.3390/nu17243825)
Supplement: Supplementary file 1 [file nutrients-17-03825-s001.zip › nutrients-3998114-supplementary.pdf]

Supplementary Table S1. Sociodemographic characteristics of mothers.

Socio-demographic characteristics	N	%
Mothers		
**Age, years**		
25 – 29	9	32.1
30 – 34	10	35.7
35 – 39	7	25.0
40 – 44	2	7.2
**Race**		
Black or African American	7	25.0
White or Caucasian	20	71.4
Mixed	1	3.6
**Ethnicity**		
Non-Hispanic	23	82.1
Hispanic	5	17.9
**Educational level completed**		
High school	1	3.6
Some college	2	7.1
2 or 4-year degree	11	39.3
Graduate degree	14	50.0
**Income group**		
Low-income	13	46.4
High income	12	42.9
3.00	3	10.7
**Household size**		
1 - 3	16	69.6
4 - 5	7	30.4
**Time (months) household could maintain current living standard if income was lost**		
< 1	2	7.1
1 - 2	5	17.9
3 - 6	5	17.9
7 - 12	4	14.3
> 12	12	42.8
**Number of adults who earn income in the household**		
1	11	39.3
2	17	60.7
Children		
**Age; months**		
0 - 5.9	8	28.6
6 - 11.9	13	46.4
12 - 17.9	6	21.4
18 – 24.0	1	3.6
**Sex**		
Boy	14	50.0
Girl	14	50.0
